# New insulin delivery devices and glycemic outcomes in young patients with type 1 diabetes: a protocol for a systematic review and meta-analysis

**DOI:** 10.1186/s13643-019-1171-9

**Published:** 2019-11-04

**Authors:** Tiago Jeronimo Dos Santos, Juan de Mata Donado Campos, Cristina Alexandra Fraga Medin, Jesús Argente, Fernando Rodríguez-Artalejo

**Affiliations:** 1 Department of Preventive Medicine and Public Health. School of Medicine, Universidad Autónoma de Madrid/IdiPAZ, Madrid, Spain; 2 Departments of Pediatrics & Pediatric Endocrinology, Hospital Infantil Universitario Niño Jesús. Research Institute “La Princesa”, Madrid, Spain; 3 Centro de Investigación Biomédica en Red de Epidemiología y Salud Pública (CIBERESP), Instituto de Salud Carlos III, Madrid, Spain; 4 Biblioteca Nacional de Ciencias de la Salud, Instituto de Salud Carlos III, Madrid, Spain; 5 Department of Pediatrics. School of Medicine, Universidad Autónoma de Madrid, Madrid, Spain; 6 Centro de Investigación Biomédica en Red de Obesidad y Nutrición (CIBEROBN), Instituto de Salud Carlos III, Madrid, Spain; 7 IMDEA Food Institute, CEIUAM+CSI, Madrid, Spain

**Keywords:** Insulin pump, Continuous subcutaneous insulin infusion, Multiple daily injections, Health inequity, Type 1 diabetes

## Abstract

**Background:**

Optimal type 1 diabetes mellitus (T1D) care requires lifelong appropriate insulin treatment, which can be provided either by multiple daily injections (MDI) of insulin or by continuous subcutaneous insulin infusion (CSII). An increasing number of trials and previous systematic reviews and meta-analyses (SRMA) have compared both CSII and MDI but have provided limited information on equity and fairness regarding access to, and the effect of, those insulin devices. This study protocol proposes a clear and transparent methodology for conducting a SRMA of the literature (1) to assess the effect of CSII versus MDI on glycemic and patient-reported outcomes (PROs) among young patients with T1D and (2) to identify health inequalities in the use of CSII.

**Methods:**

This protocol was developed based on the Preferred Reporting Items for Systematic Reviews and Meta-Analysis Protocols (PRISMA-P), the PRISMA-E (PRISMA-Equity 2012 Guidelines), and the Cochrane Collaboration Handbook. We will include randomized clinical trials and non-randomized studies published between January 2000 and June 2019 to assess the effectiveness of CSII versus MDI on glycemic and PROs in young patients with T1D. To assess health inequality among those who received CSII, we will use the PROGRESS framework. To gather relevant studies, a search will be conducted in MEDLINE, EMBASE, Cochrane Central Register of Controlled Trials (CENTRAL), the Cochrane Database of Systematic Reviews, and the Health Technology Assessment (HTA) database. We will select studies that compared glycemic outcomes (the glycosylated hemoglobin values, severe hypoglycemia episodes, diabetic ketoacidosis events, and/or time spent in range or in hyper-hypoglycemia), and health-related quality of life, as a PRO, between therapies. Screening and selection of studies will be conducted independently by two researchers. Subgroup analyses will be performed according to age group, length of follow-up, and the use of adjunctive technological therapies that might influence glycemic outcomes.

**Discussion:**

Studies of the average effects of CSII versus MDI may have not assessed their impact on health equity, as some intended populations have been excluded. Therefore, this study will address health equity issues when assessing effects of CSII. The results will be published in a peer-review journal. *Ethics* approval will not be needed.

**Systematic review registration:**

PROSPERO CRD42018116474

## Background

Optimal type 1 diabetes mellitus (T1D) care requires lifelong appropriate insulin treatment that can be provided by either multiple daily injections (MDI) of insulin or by a continuous subcutaneous insulin infusion (CSII) pump [1]. Over the last years, the use of CSII has increased substantially among pediatric patients [1]. However, the selection of CSII versus MDI might have not been based only on clinical indications (e.g., elevated glycosylated hemoglobin and higher hypoglycemia rate), but also could have been influenced by social factors, such as the place of residence and socioeconomic status, which may have led to health inequalities [1–3].

Meeting glycemic targets is a challenging task in young patients with T1D; thus, new insulin delivery systems represent an opportunity to improve glycemic control, to promote patient-centered decisions, and to reduce the burden of diabetes care [4, 5]. Although an increasing number of trials has assessed whether the CSII is more effective than the intensive insulin therapy with syringe and/or pen [6–13], previous systematic reviews and meta-analyses (SRMA) of trials have not reported adequate information concerning equity and fairness in treatment selection [14–17].

Given the greater difficulty for good glycemic control in patients/families with lower health literacy and poor access to some healthcare resources, it is possible that the absolute benefit of CSII would be greater in those with lower socioeconomic status [18]. However, we do not know if they have the chance to participate and benefit from this intervention. In addition, there might exist several barriers for patient access and/or maintenance using CSII, and only a few studies (e.g., diabetes registries) have investigated the role of unequal health care access and social disparities on glycemic outcomes [2, 19, 20]. In consequence, SRMAs with an equity lens could assess whether unequal benefits across sociodemographic population groups could contribute to worsening health inequalities in T1D management [21–23].

Therefore, this paper aims to report a standardized and transparent methodology for conducting a SRMA of the literature (1) to assess the effectiveness of using CSII versus MDI on glycemic (glycosylated hemoglobin, severe hypoglycemia, diabetes ketoacidosis and glycemic variability) and patient-related outcomes among young patients with T1D and (2) to identify health inequalities for those who use CSII.

## Methods

### Review design

This protocol was developed based on the Preferred Reporting Items for Systematic Reviews and Meta-Analysis Protocols (PRISMA-P) [24] and was registered and published on PROSPERO international prospective register of systematic reviews (registration number CRD42018116474). The Cochrane Collaboration Handbook [25] will also be used to guide the review methods, and PRISMA-E (PRISMA-Equity 2012) Guidelines [26] to elaborate the final report. To perform the SRMA, we will include randomized clinical trials (RCT) and non-randomized studies (NRS)—which cover diabetes registries and longitudinal studies—that compared the clinical effectiveness of CSII versus MDI in youths with T1D.

### Data sources and search strategy

The bibliographic search will be conducted from January 2000 to June 2019 in MEDLINE (via PubMed), EMBASE, Cochrane Central Register of Controlled Trials (CENTRAL), the Cochrane Database of Systematic Reviews, and the Health Technology Assessment (HTA) Database. We will also carry out a handsearch of the previous reviews and the bibliography from the original articles for additional references, as well as of the gray literature focusing on abstracts from diabetes associations and conference proceedings, and from technical reports (research and governmental agencies). Search will use standardized subject terms and will be conducted by a librarian with the input from the principal investigator, using Boolean operators for MEDLINE, EMBASE, CENTRAL, and HTA database. The final search strategy will have no restrictions based on language or publication status (see Additional file 1).

### Eligibility criteria

We will select studies that compared the use of CSII with MDI and evaluated any of the following glycemic outcomes: glycosylated hemoglobin (HbA_1c_, percentage), the incidence of hypoglycemia episodes [e.g., severe, serious and/or nocturnal], diabetic ketoacidosis (DKA) events, and/or time spent in range or in hyper-hypoglycemia. Studies that mentioned health-related quality of life (HRQoL) as a PRO will also be selected. Specifically, the studies must meet the following selection criteria: (1) to be conducted with children and adolescents (under 20 years of age), (2) exclusively on patients with T1D, (3) designed as RCT or NRS, and (4) to have reported any of the outcomes of interest: HbA_1c_, hypoglycemia, DKA, time in range or in hyper-hypoglycemia, and HRQoL. Bi-hormonal or dual-hormone closed-loop systems that deliver glucagon in addition to insulin will not be included.

### Equity analysis

To explore equity in CSII, we will use indicators of social disadvantages defined by PROGRESS [27]. The acronym PROGRESS is a framework to guide data extraction to relate the outcomes with equity of access to an intervention, according to “*place of residence*” (residing in a high- or low-to-middle-income country, as per the World Bank database), “*race, ethnicity, culture and language*” (racial, ethnical, and cultural background, when the majority of the groups include belonging to a distinctive group who shares origin, culture, traditions, and language through generations), “*occupation*” (parental patterns of work that favor proper maintenance of a therapy or not), “*gender/sex*” (sex refers to identify sex distribution when recommended each therapy), “*religion*” (religious affiliation, spiritual beliefs, or values that promote better access to health services), “*education*” (assumes that high parental educational level, or health literacy and numeracy, is an advantage), “*socioeconomic status*” (access to resources and privilege with greater household wealth, as an advantage), and “*social capital*” (benefits obtained by individuals due to their social relationships, as an advantage).

For each factor of inequality, we hypothesized different social gradients: (1) a positive gradient, when better glycemic outcomes are found in more socially advantaged groups; (2) a negative gradient, when better outcomes are found in less advantaged groups; and (3) a neutral gradient, when no significant differences exist between groups. The results will be summarized with the aid of a harvest plot, which is a graphical technique that helps to illustrate a narrative synthesis [28].

### Study selection and data extraction

Two reviewers will work independently to check eligibility of studies (title and abstract and, if needed, full-text) and extract the appropriate information in full-text articles. Disagreements will be resolved by consensus. Assessment of eligibility and its inclusion will be conducted according to the indications of the PRISMA statement. Data to be extracted from articles include the year of publication, country, study design and period of data collection, baseline characteristics of participants, interventions and comparators, factors of inequalities at baseline, and outcomes (Tables 1 and 2).

**Table 1:** Table of evidence with main characteristics of the included studies

Reference	Design and registration details; founding	Country	Year of baseline data collection	N and clinical characteristics	Setting	Type of diabetes-related technology	Type of conventional treatment comparator	Inequality assessed from baseline characteristics	Outcomes	Length of follow-up
Authors, year of publication, name of the study	Study design/registration number/founding	Country or region	Year	Number of patients assigned and that received each treatment (CSII:MDI); Sex (M:F); Age; Baseline characteristics of participants (including duration of the disease, baseline HbA_1c_ [mean % (SD)] and HRQoL assessment tool); Other definition or comment	Community/clinical based research	Continuous subcutaneous insulin infusion (CSII), including the use of adjunctive glucose monitors: model of devices and insulin	Multiple daily injections (MDI): injections and insulins	A. Place of residence B. Race, ethnicity, culture and language C. Occupation D. Sex E. Religion F. Education G. Socioeconomic status H. Social capital	1. HbA_1c_ at the end of the study: CSII versus MDI [mean % (SD)], sig 2. Total number of hypoglycemic episodes: CSII versus MDI, sig 3. Number of patients with a frequency of ≥ 1 Ketoacidosis episode: CSII versus MDI, sig 4. Glycemic variability: % of time in range, hypo and/or hyperglycemia: CSII versus MDI, sig 5. HRQoL score (±SD) at the end of the study: CSII vs. MDI, sig	Duration of follow

*CSII* continuous subcutaneous insulin infusion, *MDI* multiple daily injection, *M* male, *F* female, *HbA*_*1c*_ glycosylated hemoglobin, *SD* standard deviation, *sig* significance, *HRQoL* health-related quality of life

**Table 2:**
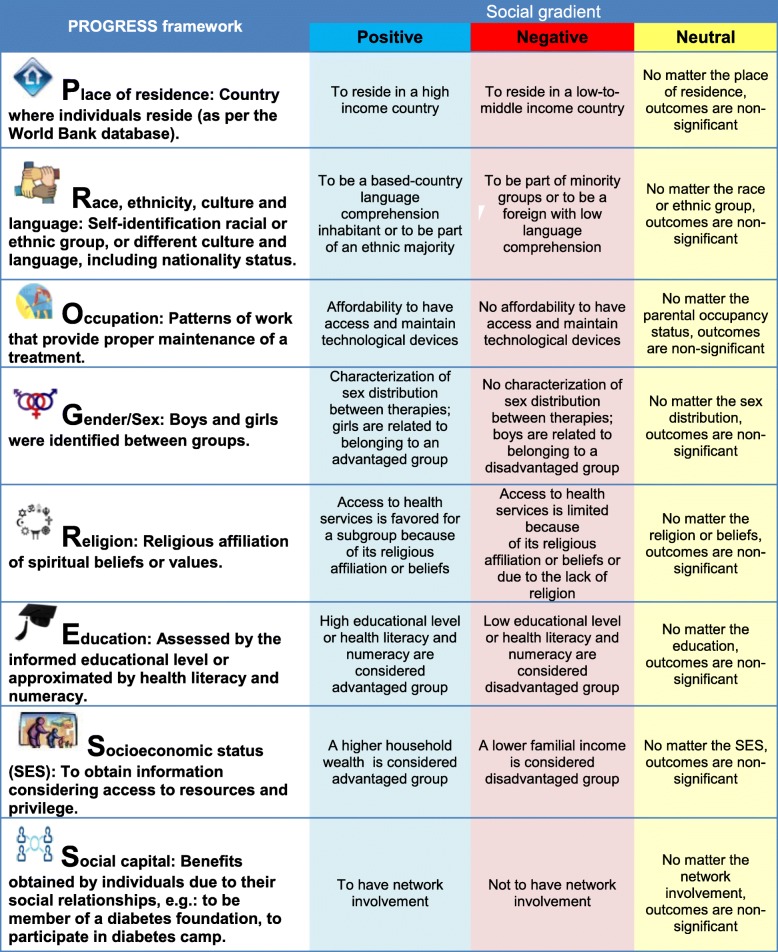
PROGRESS framework to guide health equity data extraction on type 1 diabetes

The glycemic endpoints include (1) the mean value of HbA_1c_ (percentage), assessed preferably at the end of the study, (2) the number of serious, severe and/or nocturnal hypoglycemia episodes [≤ 3.0 mmol/L (54 mg/dL) or an event associated with severe cognitive impairment (including coma and convulsions) requiring assistance], (3) the number of patients with ≥ 1 DKA event, and (4) the percentage of time spent in range [percentage of readings in the glycemic range of 3.9–10.0 mmol/L (70–180 mg/dL) per unit of time] or in hypo [< 3.9 mmol/L (< 70 mg/dL)] and hyperglycemia [> 10 mmol/L (> 180 mg/dL)] [23, 29–32]. PRO will be captured with the HRQoL questionnaires. When necessary, authors of eligible studies will be contacted to provide additional information.

### Assessment of risk of bias

Two reviewers will independently assess the risk of bias of each study using two different tools: the Cochrane Risk of Bias form RCT and the RTI Item Bank for NRS [33, 34]. A review of only RCT may provide insufficient information on vulnerable subpopulations. Still, the inclusion of NRS may increase the challenges in establishing causal inference because they are at greater risk of bias than RCT, resulting from confounding by indication and selection bias. In contrast, threats to validity from performance and detection bias, and to precision from the inadequate sample size, should not differ markedly between RCT and NRS (although some features such as blinding of assessors that protect against detection bias are more likely in experimental designs than in observational studies). By including NRS (mainly registries), we may capture valuable information on the intended population for whom CSII is preferred, because registries are larger, studied over a longer time, and may better reflect all subgroups of patients and routine clinical practice [3].

### Statistical analysis

We will summarize the main characteristics of selected studies, including the study’s objectives and design, characteristics of study participants, intervention and comparator, inclusion of PROGRESS categories, and outcomes (Tables 1 and 2). Effects across the studies will be summarized with (1) the pooled mean difference for HbA_1c_; (2) the pooled rate ratio for hypoglycemia; (3) the pooled risk ratio for DKA; (4) the mean difference in percentage of time that blood glucose concentration remained in target range, in hypo- or in hyperglycemia; and (5) the pooled standardized mean difference (SMD) for quality of life outcomes, with their 95% confidence interval (CI), calculated with inverse variance random effects models to incorporate the level of heterogeneity found across studies [25, 35]. The effect size of the SMD will be classified as small (0.1–0.3), medium (0.3–0.6) or large (≥ 0.6) [36]. Heterogeneity among studies will be assessed with the *I*^2^ statistic, whose values will be classified as follows: no relevant heterogeneity (0–25%), moderate heterogeneity (25–50%), and substantial heterogeneity (> 50%) [37]. Meta-analyses will be performed separately for RCTs and NRS when data are available for at least two studies with comparable results. For equity outcomes, results will be summarized as a narrative synthesis [28]. Publication bias will be evaluated graphically using a funnel plot and also with the method of Egger et al. [37]. The strength of the body of evidence will be assessed using the Grading of Recommendations Assessment, Development and Evaluation (GRADE) tool [38].

### Subgroup analysis

Subgroup analyses will be performed based on age group, length of follow-up, and the use of adjunctive technological therapies that might directly improve glycemic outcomes.

### Sensitivity analysis

The analyses will be repeated after exclusion of studies with a high risk of bias, and separately for RCT and NRS.

## Discussion

Given the increase of worldwide incidence of T1D, the wider use of the CSII pump among some specific socioeconomic and demographic groups, and the lack of evidence of its superiority when compared with the conventional therapy using MDI, there is a need to critically assess the rise of inequalities in treatment selection [39]. Furthermore, the inclusion of PRO captured by health-related quality of life questionnaires will contribute to a complete diabetes measures portfolio [40]. Hence, the assessment of the effects of CSII versus MDI on glycemic outcomes, across social factors defined by PROGRESS, may contribute better to understand their impact on health equity [12, 16, 41, 42].

A major issue will probably be the limited data reported in the reviewed studies on the PROGRESS factors. For this reason, supplementary information will also be gathered from authors of the included studies. We are aware that the lack of important published information on equity may be a limitation of our review.

The results of an equity-oriented SRMA may yield an opportunity to discuss not only the effects of such interventions on glycemic endpoints, but also the existing gap of information in the included studies regarding social inequities; it will pave the way to use those results to orient clinical practice, equity-based research, and health policy formulation.

## Supplementary information

**Additional file 1.** Search Strategies.

## Data Availability

Not applicable

## Abbreviations

CSII: Continuous subcutaneous insulin infusion
DKA: Diabetes ketoacidosis
GRADE: Grading of Recommendations Assessment, Development and Evaluation
HbA_1c_: Glycosylated hemoglobin
HRQoL: Health-related quality of life
HTA: Health Technology Assessment
MDI: Multiple daily injections
NRS: Non-randomized studies
PRISMA-E: Preferred Reporting Items for Systematic Reviews and Meta-Analysis – Equity Report
PRISMA-P: Preferred Reporting Items for Systematic Reviews and Meta-Analysis Protocols
PRO: Patient-related outcome
PROGRESS: Place of residence, race/ethnicity/culture/language, occupation, gender/sex, religion, education, socioeconomic status, and social capital
RCT: Pandomized clinical trials
SMD: Standardized mean difference
SRMA: Systematic review and meta-analysis
T1D: Type 1 diabetes mellitus
