# Supplementary material for: New insulin delivery devices and glycemic outcomes in young patients with type 1 diabetes: a protocol for a systematic review and meta-analysis
Source: Syst Rev. 2019 Nov 4;8:259. doi: 10.1186/s13643-019-1171-9 (PMC6829915; doi:10.1186/s13643-019-1171-9)
Supplement: Supplementary file 1 — Additional file 1. Search Strategies. [file 13643_2019_1171_MOESM1_ESM.docx]

**Search Strategies:**

***Medline (via PubMed)***

| **Search** | **Query** | **Items found** |
| --- | --- | --- |
| #31 | Search (#27 AND #28) Filters: Publication date from 2000/01/01 to 2019/06/30; Humans | 391 |
| #30 | Search (#27 AND #28) Filters: Humans | 446 |
| #29 | Search (#27 AND #28) | 536 |
| #28 | Search ((((((((((infant OR infan* OR child OR children OR schoolchild* OR schoolchild OR school child OR school child* OR kid OR kids OR toddler* OR adolescent OR boy OR girl OR minors OR minors* OR underag* OR under ag* OR juvenil* OR youth* OR kindergar* OR puberty OR puber* OR pubescen* OR prepubescen* OR prepuberty* OR pediatrics OR pediatric* OR paediatric* OR peadiatric* OR schools OR nursery school* OR preschool* OR pre school* OR primary school* OR secondary school* OR elementary school* OR elementary school OR highschool* OR highschool* OR school age OR school age OR school age* OR schoolage* OR infancy OR schools, nursery))) NOT fetus) NOT newborn) NOT neonatal))))) | [4687061](https://www.ncbi.nlm.nih.gov/pubmed/?cmd=HistorySearch&querykey=60) |
| #27 | Search (#19 AND #26) | 1096 |
| #26 | Search (#20 OR #21 OR #22 OR #23 OR #24 OR #25) | [18225](https://www.ncbi.nlm.nih.gov/pubmed/?cmd=HistorySearch&querykey=57) |
| #25 | Search ((“short acting insulin”[tiab] or "regular insulin" or "isophane insulin" or "human insulin" or "humulin")) OR “rapid acting insulin”[tiab] or "aspart" or "insulin aspart" or "Insulin-Aspart" or "NovoLog" or "Novorapid" or "lispro" or "insulin lispro" or "lyspro" or "Humalog Kwikpen" or "humalog" or "apidra" or "insulin apidra"))) | [10123](https://www.ncbi.nlm.nih.gov/pubmed/?cmd=HistorySearch&querykey=56) |
| #24 | Search ((basal*[tiab] AND bolus[tiab] AND (injection*[tiab] OR regime*[tiab] OR routine*[tiab] OR system*[tiab]))) | [1959](https://www.ncbi.nlm.nih.gov/pubmed/?cmd=HistorySearch&querykey=55) |
| [#2](https://www.ncbi.nlm.nih.gov/pubmed/advanced)3 | Search MDI[tiab] | [3457](https://www.ncbi.nlm.nih.gov/pubmed/?cmd=HistorySearch&querykey=25) |
| [#2](https://www.ncbi.nlm.nih.gov/pubmed/advanced)2 | Search (((“multiple injection”[tiab] or “multiple injections”[tiab] or “multiple insulin”[tiab] or “multiple regime”[tiab] or “multiple regimes”[tiab] or “multiple routine”[tiab] or “multiple routines”[tiab])))) | [2837](https://www.ncbi.nlm.nih.gov/pubmed/?cmd=HistorySearch&querykey=24) |
| [#2](https://www.ncbi.nlm.nih.gov/pubmed/advanced)1 | Search (((“multiple dose injection”[tiab] or “multiple dose injections”[tiab] or “multiple dose insulin”[tiab] or “multiple dose regime”[tiab] or “multiple dose regimes”[tiab] or “multiple dose routine”[tiab] or “multiple dose routines”[tiab])))) | [61](https://www.ncbi.nlm.nih.gov/pubmed/?cmd=HistorySearch&querykey=23) |
| [#2](https://www.ncbi.nlm.nih.gov/pubmed/advanced)0 | Search ((((“multiple daily injection”[tiab] or “multiple daily injections”[tiab] or “multiple daily insulin”[tiab] or “multiple daily regime”[tiab] or “multiple daily regimes”[tiab] or “multiple daily routine”[tiab] or “multiple daily routines”[tiab])))) | [1010](https://www.ncbi.nlm.nih.gov/pubmed/?cmd=HistorySearch&querykey=22) |
| [#19](https://www.ncbi.nlm.nih.gov/pubmed/advanced) | Search ((#9 AND #18)) | 4238 |
| [#18](https://www.ncbi.nlm.nih.gov/pubmed/advanced) | Search ((((#10 OR #11 OR #12 OR #13 OR #14 OR #15 OR #16 OR #17))) | 11793 |
| [#17](https://www.ncbi.nlm.nih.gov/pubmed/advanced) | Search ((((("animas" or vibe) AND (pump* or infus* or system*)))))) | [151](https://www.ncbi.nlm.nih.gov/pubmed/?cmd=HistorySearch&querykey=17) |
| [#16](https://www.ncbi.nlm.nih.gov/pubmed/advanced) | Search (((“veo pump” or “veo pumps”))) | [26](https://www.ncbi.nlm.nih.gov/pubmed/?cmd=HistorySearch&querykey=16) |
| [#15](https://www.ncbi.nlm.nih.gov/pubmed/advanced) | Search ((((paradigm* AND (veo or pump*)))))) | [512](https://www.ncbi.nlm.nih.gov/pubmed/?cmd=HistorySearch&querykey=15) |
| [#14](https://www.ncbi.nlm.nih.gov/pubmed/advanced) | Search ((("minimed" or "paradigmaveo"))) | [312](https://www.ncbi.nlm.nih.gov/pubmed/?cmd=HistorySearch&querykey=14) |
| [#13](https://www.ncbi.nlm.nih.gov/pubmed/advanced) | Search ((((accu-chek[tiab] or cellnovo[tiab] or “dana diabecare”[tiab] or omnipod[tiab]))))) | [245](https://www.ncbi.nlm.nih.gov/pubmed/?cmd=HistorySearch&querykey=13) |
| [#12](https://www.ncbi.nlm.nih.gov/pubmed/advanced) | Search (((“subcutaneous insulin”[tiab] or CSII[tiab])))) | [3261](https://www.ncbi.nlm.nih.gov/pubmed/?cmd=HistorySearch&querykey=12) |
| [#11](https://www.ncbi.nlm.nih.gov/pubmed/advanced) | Search (((“pump therapy”[tiab] or “pump therapies”[tiab] or “pump treatment”[tiab] or “pump treatments”[tiab])))) | [1451](https://www.ncbi.nlm.nih.gov/pubmed/?cmd=HistorySearch&querykey=11) |
| [#10](https://www.ncbi.nlm.nih.gov/pubmed/advanced) | Search ((“insulin pump”[tiab] or “insulin pumps”[tiab] or “insulin infusion”[tiab] or “insulin infuse”[tiab] or “insulin infused”[tiab] or “insulin deliver”[tiab] or “insulin delivery”[tiab])) | [9724](https://www.ncbi.nlm.nih.gov/pubmed/?cmd=HistorySearch&querykey=10) |
| [#9](https://www.ncbi.nlm.nih.gov/pubmed/advanced) | Search ((#1 OR #2 OR #3 OR #4 OR #5 OR #6 OR #7 OR #8)) | [80050](https://www.ncbi.nlm.nih.gov/pubmed/?cmd=HistorySearch&querykey=9) |
| [#8](https://www.ncbi.nlm.nih.gov/pubmed/advanced) | Search ((dm1[tiab] or “dm 1”[tiab] or t1dm[tiab] or “t1 dm”[tiab] or t1d[tiab] or iddm[tiab])) | [19663](https://www.ncbi.nlm.nih.gov/pubmed/?cmd=HistorySearch&querykey=8) |
| [#7](https://www.ncbi.nlm.nih.gov/pubmed/advanced) | Search ((“insulin dependent”[tiab] or insulindepend*[tiab])) | [28768](https://www.ncbi.nlm.nih.gov/pubmed/?cmd=HistorySearch&querykey=7) |
| [#6](https://www.ncbi.nlm.nih.gov/pubmed/advanced) | Search ((“brittle diabetic”[tiab] or “diabetic juvenile”[tiab] or “diabetic pediatric”[tiab] or “diabetic paediatric”[tiab] or “diabetic early”[tiab] or “diabetic labile”[tiab] or “diabetic acidosis”[tiab] or “diabetic sudden onset”[tiab])) | [359](https://www.ncbi.nlm.nih.gov/pubmed/?cmd=HistorySearch&querykey=6) |
| [#5](https://www.ncbi.nlm.nih.gov/pubmed/advanced) | Search ((“diabetic brittle”[tiab] or “juvenile diabetic”[tiab] or “pediatric diabetic”[tiab] or “paediatric diabetic”[tiab] or “early diabetic”[tiab] or “labile diabetic”[tiab] or “acidosis diabetic”[tiab] or “sudden onset diabetic”[tiab])) | [1401](https://www.ncbi.nlm.nih.gov/pubmed/?cmd=HistorySearch&querykey=5) |
| [#4](https://www.ncbi.nlm.nih.gov/pubmed/advanced) | Search ((“brittle diabetes”[tiab] or “diabetes juvenile”[tiab] or “diabetes pediatric”[tiab] or “diabetes paediatric”[tiab] or “diabetes early”[tiab] or “diabetes ketosis”[tiab] or “diabetes labile”[tiab] or “diabetes acidosis”[tiab] or “diabetes sudden onset”[tiab])) | [323](https://www.ncbi.nlm.nih.gov/pubmed/?cmd=HistorySearch&querykey=4) |
| [#3](https://www.ncbi.nlm.nih.gov/pubmed/advanced) | Search ((“diabetes brittle”[tiab] or “juvenile diabetes”[tiab] or “pediatric diabetes”[tiab] or “paediatric diabetes”[tiab] or “early diabetes”[tiab] or “ketosis diabetes”[tiab] or “labile diabetes”[tiab] or “acidosis diabetes”[tiab] or “sudden onset diabetes”[tiab])) | [2691](https://www.ncbi.nlm.nih.gov/pubmed/?cmd=HistorySearch&querykey=3) |
| [#2](https://www.ncbi.nlm.nih.gov/pubmed/advanced) | Search ((“diabetic type 1”[tiab] OR “type 1 diabetic”[tiab] OR “diabetic type i”[tiab] OR “type i diabetic”[tiab] OR “diabetic type1”[tiab] OR “type1 diabetic”[tiab] OR “diabetic typei”[tiab] OR “typei diabetic”[tiab])) | [7149](https://www.ncbi.nlm.nih.gov/pubmed/?cmd=HistorySearch&querykey=2) |
| [#1](https://www.ncbi.nlm.nih.gov/pubmed/advanced) | Search ((((“diabetes type 1”[tiab] OR “type 1 diabetes”[tiab] OR “diabetes type i”[tiab] OR “type i diabetes”[tiab] OR “diabetes type1”[tiab] OR “type1 diabetes”[tiab] OR “diabetes typei”[tiab] OR “typei diabetes”[tiab])))) | [42483](https://www.ncbi.nlm.nih.gov/pubmed/?cmd=HistorySearch&querykey=1) |

***Embase (via Elsevier)***

**'insulin dependent diabetes mellitus'**/exp AND (**'insulin pump'**/mj OR **'accu chek spirit'** OR **'d-tron'** OR **'d-tronplus'** OR **'dana diabecare'** OR **'deltec cozmo'** OR **'h-tron plus'** OR **'h-tronplus'** OR **'minimed 508'** OR **'minimed 530g pump'** OR **'minimed paradigm 508'** OR **'minimed paradigm revel'** OR **'minimed paradigm veo'** OR **'minimed paradigm 512'** OR **'minimed paradigm 712'** OR **'omnipod'** OR **'onetouch ping'** OR **'v-go (device)'** OR **'vibe (device)'** OR **'zone (device)'** OR **'insulin infusion system'** OR **'insulin pump'** OR **'insulin pump, device (physical object)'** OR **'pump, infusion, insulin'** OR **'pump, insulin'** OR **'t:slim x2'**) AND (**'insulin injection pen'**/exp OR **'autopen (insulin injection pen)'** OR **'flexpen'** OR **'flextouch'** OR **'humapen'** OR **'humapen ergo'** OR **'humapen luxura hd'** OR **'humapen memoir'** OR **'humapen savvio'** OR **'innolet'** OR **'innovo (device)'** OR **'kwikpen'** OR **'novofine'** OR **'novopen 4'** OR **'novopen 5'** OR **'novopen echo'** OR **'opticlik'** OR **'solostar'** OR **'insulin injection pen'** OR **'insulin pen'**) AND **([adolescent]/lim** OR **[child]/lim** OR **[infant]/lim** OR **[preschool]/lim** OR **[school]/lim** OR **[young adult]/lim)**

#4 **[adolescent]/lim** OR **[child]/lim** OR **[infant]/lim** OR **[preschool]/lim** OR **[school]/lim** OR **[young adult]/lim -- 17**

#3 **'insulin injection pen'**/exp OR **'autopen (insulin injection pen)'** OR **'flexpen'** OR **'flextouch'** OR **'humapen'** OR **'humapen ergo'** OR **'humapen luxura hd'** OR **'humapen memoir'** OR **'humapen savvio'** OR **'innolet'** OR **'innovo (device)'** OR **'kwikpen'** OR **'novofine'** OR **'novopen 4'** OR **'novopen 5'** OR **'novopen echo'** OR **'opticlik'** OR **'solostar'** OR **'insulin injection pen'** OR **'insulin pen' -- 53**

#2 **'insulin pump'**/mj OR **'accu chek spirit'** OR **'d-tron'** OR **'d-tronplus'** OR **'dana diabecare'** OR **'deltec cozmo'** OR **'h-tron plus'** OR **'h-tronplus'** OR **'minimed 508'** OR **'minimed 530g pump'** OR **'minimed paradigm 508'** OR **'minimed paradigm revel'** OR **'minimed paradigm veo'** OR **'minimed paradigm 512'** OR **'minimed paradigm 712'** OR **'omnipod'** OR **'onetouch ping'** OR **'v-go (device)'** OR **'vibe (device)'** OR **'zone (device)'** OR **'insulin infusion system'** OR **'insulin pump'** OR **'insulin pump, device (physical object)'** OR **'pump, infusion, insulin'** OR **'pump, insulin'** OR **'t:slim x2' – 4,848**

#1 **'insulin dependent diabetes mellitus'**/exp – 111,599

***CENTRAL (via Cochrane Library)***

#1 MeSH descriptor: [Insulins] explode all trees and with qualifier(s): [administration & dosage - AD, therapeutic use - TU] -- 4223

#2 MeSH descriptor: [Insulin Infusion Systems] explode all trees -- 605

#3 MeSH descriptor: [Injections, Subcutaneous] explode all trees—4257

#4 {OR #2-#3} – 4756

#5 MeSH descriptor: [Diabetes Mellitus, Type 1] explode all trees – 4906

#6 (MULTIPLE DAILY INJECTION):ti,ab,kw – 875

#7 #5 AND #4 OR #6 AND #1 – 719

#8 (child or child* or young or young* or minors or underag* or juvenil or youth or pediatric* or peadiatric*):ti,ab NOT fetus NOT neonatal NOT newborn -- 147900

#9 #7 AND #8 – 118

with Cochrane Library publication date from Jan 2000 to Jun 2019

***HTA Database (via CRD Database)***

#1 MeSH DESCRIPTOR Diabetes Mellitus, Type 1 EXPLODE ALL TREES -- 312

#2 MeSH DESCRIPTOR Insulin Infusion Systems EXPLODE ALL TREES – 71

#3 MeSH DESCRIPTOR Injections, Subcutaneous EXPLODE ALL TREES – 127

#4 (MeSH DESCRIPTOR Diabetes Mellitus, Type 1 EXPLODE ALL TREES) OR (MeSH DESCRIPTOR Insulin Infusion Systems EXPLODE ALL TREES) OR (MeSH DESCRIPTOR Injections, Subcutaneous EXPLODE ALL TREES) IN HTA FROM 2000 TO 2019 -- 33
